# Intronic Variants in the *MSH2* (rs2303426 and rs10179950) and *PMS2* (rs2286681 and rs62456178) Genes Are Not Associated with Colorectal Cancer in Mexican Patients

**DOI:** 10.3390/genes15111380

**Published:** 2024-10-26

**Authors:** Manuel Alejandro Rico-Méndez, Anna Guadalupe López-Ceballos, José Miguel Moreno-Ortiz, María de la Luz Ayala-Madrigal, Melva Gutiérrez-Angulo, Ruth Ramírez-Ramírez, Mirna Gisel González-Mercado, Anahí González-Mercado

**Affiliations:** 1 Doctorado en Genética Humana e Instituto de Genética Humana “Dr. Enrique Corona Rivera”, Departamento de Biología Molecular y Genómica, Centro Universitario de Ciencias de la Salud, Universidad de Guadalajara, Guadalajara 44340, Mexico; manuel.rico8557@alumnos.udg.mx (M.A.R.-M.); miguel.moreno@academicos.udg.mx (J.M.M.-O.); luz.ayala@academicos.udg.mx (M.d.l.L.A.-M.); melva.gutierrez@academicos.udg.mx (M.G.-A.); 2 Departamento de Ciencias de la Salud, Centro Universitario de los Altos, Universidad de Guadalajara, Tepatitlán de Morelos 47600, Mexico; 3 Departamento de Biología Celular y Molecular, Centro Universitario de Ciencias Biológicas y Agropecuarias, Universidad de Guadalajara, Zapopan 45200, Mexico; ruth.ramirez@academicos.udg.mx; 4 Escuela de Medicina y Ciencias de la Salud, Tecnológico de Monterrey, Campus Guadalajara, Zapopan 45138, Mexico; gisel.gonzalez@tec.mx

**Keywords:** colorectal cancer, *MSH2*, *PMS2*, rs2303426, rs10179950, rs2286681, rs62456178

## Abstract

Background/Objectives: In the origin and development of colorectal cancer (CRC), a global public health problem, a dysfunction mismatch repair system appears to be a key factor. The objective was to determine the association of intronic variants in the *MSH2* and *PMS2* genes with CRC in Mexican patients. Methods: Blood samples of 143 CRC patients and 146 reference individuals were genotyped through TaqMan^®^ Genotyping Assays. Genotypic and allelic frequencies were determined by direct counting. To compare genotypic and allelic distributions, the chi-square test was used. For the association analysis, the risks of alleles and genotypes were estimated by odds ratio with 95% confidence intervals. Haplogroups were inferred with a Bayesian algorithm. Linkage disequilibrium was measured using D’ and r^2^ with Arlequin v3.5.2. The in silico analysis was carried out using the SpliceAI, UCSC, JASPAR and TRRUST platforms. All statistical analyses were performed with SPSS v29.0.2.0. Results: In the CRC group, the mean age was 58.2 ± 14.7 years and 60.8% were men. No variant was associated with CRC or implicated in gene post-replicative processing. Linkage disequilibrium was observed for loci rs2303426 and rs10179950 in *MSH2* and for loci rs2286681 and rs62456178 in *PMS2*. Conclusions: The genotypic and allelic frequencies of the four variants are reported for the first time in Mexican patients with CRC. No association was found between gene variants and risk for CRC but there was a strong linkage disequilibrium between the loci of both *MSH2* and *PMS2* genes. None of the variants showed a possible repercussion on splicing.

## 1. Introduction

Colorectal cancer (CRC), a major public health problem worldwide, ranked third in incidence and second in mortality in 2022 according to the World Health Organization (WHO) and International Agency for Research on Cancer (IARC) through the Global Cancer Observatory. Moreover, both its incidence and mortality rates will likely double by 2050 with similar trends for Mexico [1]. Among the etiopathogenic mechanisms implied in this disease, alterations in the mismatch repair system (MMR) stand out [2].

The MMR is responsible for correcting base pairing mistakes that occur during DNA replication and thereby contributes to maintaining genomic stability [3]. In humans, this system generally acts via the formation of heterodimers. When the mismatch is caused by a single nucleotide variant (SNV) or a 1–2 pb insertion/deletion (INDEL), it is recognized by the MutSα complex formed by MutS homolog 2 (MSH2) and MutS homolog 6 (MSH6), while if a large INDEL is present, the MutSβ complex formed by MSH2 and MutS homolog 3 (MSH3) will act. Once the mismatch is recognized, the MutLα complex formed by MutL homolog 1 (MLH1) and an abundant postmeiotic segregation protein type 2 (PMS2) will ultimately regulate the cleavage of the fragment [4].

Amidst the MMR proteins, MSH2 has the highest expression, is essential for recognizing DNA mismatches and triggers cell signaling [5]. As for PMS2, it breaks the phosphodiester bond at the mismatch site, a crucial step for chain excision [4]. The MSH2 protein consists of 934 residues and is encoded by the *MSH2* gene located on 2p21-p16.3 and contains 16 exons over 80.07 kb. The PMS2 protein is a chain of 862 residues encoded by the *PMS2* gene mapped on 7p22.1 and consists of 15 exons over 38.12 kb [6].

The study of *MSH2* and *PMS2* genes is relevant for understanding the genetic basis of hereditary and sporadic CRC. Among the genes in the MMR pathway, *PMS2* presents the highest frequency of germline variants. However, variants in *MSH2* are more prevalent and are associated with a higher risk of CRC [7].

A deficient MMR pathway leads to microsatellite instability (MSI), a prognostic biomarker in CRC and other cancers with MMR alterations. Patients with MSI respond favorably to immune checkpoint inhibitors such as pembrolizumab, a programmed cell death protein 1 (PD1) inhibitor [7].

It is important to study intronic variants because they could influence the regulation of gene expression by affecting RNA splicing. Additionally, intronic sequences may contribute to numerous biological processes and can be recognized by regulatory factors, potentially leading to changes in the expression of genes involved in the development of CRC [8]. Many variants in coding and non-coding regions of MMR genes, usually with the potential to affect the function of the expressed proteins, have been reported [9]. Although the MMR has a constitutive and highly efficient expression, its functional deficiencies can lead to the accumulation of mutations and genomic instability, which in turn favor the appearance and development of cancer [10].

Different variants have been identified along the *MSH2* gene. The NM_000251.3:c.211+9 C>G (rs2303426) intronic variant (global allele frequency: C = 0.37, G = 0.63) [11] has been associated with an increased risk of developing lung [12] and gallbladder cancers [13] but not with CRC. For the NM_000251.3:c.211+811C>T (rs10179950) intronic variant (global allele frequency: G = 0.39, T = 0.61) [11], no reports were found on its association with any type of cancer. As for the *PMS2* gene, encoded on antisense DNA strand, its intronic variants NM_000535.7:c.804-164A>C (rs2286681) (global allele frequency: A = 0.63, C = 0.37) [14] and NM_000535.7:c.989-701G>A (rs62456178) (global allele frequency: G = 0.67, A = 0.33) [11] have been linked to an increased risk of developing lung cancer and head and neck carcinoma [15,16]. However, their relationship with CRC has not yet been explored.

## 2. Materials and Methods

### 2.1. Study Population

After obtaining extended informed consent, we collected peripheral blood samples from 143 patients clinically and histopathologically diagnosed with CRC (case group) and 146 apparently healthy individuals attending the blood bank (reference group) at the Civil Hospital “Juan I. Menchaca”, Guadalajara, Jalisco, Mexico. Both groups were required to be Mexican, with at least two generations of Mexican ancestry, and over the age of eighteen. Each participant underwent a survey to gather data such as age, height, weight, body mass index (BMI) and gender.

For the sample size calculation, the formula for an unmatched case–control study was used, n = (Zα/2 + Zβ)^2^ × [P1(1 − P1) + P0(1 − P0)]/(P1 − P0)^2^, with a minor allelic frequency of 0.317 in cases [11] and 0.50 in controls [12].

### 2.2. Genotyping

Genotyping of the intronic variants of the *MSH2* and *PMS2* genes was performed using TaqMan^®^ SNP Genotyping Assays (Table 1) with the ABI 7300 real-time qPCR system (Applied Biosystems, Waltham, MA, USA) and following the manufacturer’s instructions, PCR reaction mix volume for volume total 15 µL: 7.5 µL TaqMan Genotyping Master Mix (2×), 0.375 µL TaqMan genotyping assay mix (20×), 4.2 µL DNA (50 ng/µL) and 2.925 µL DNase-free water. Thermal cycling conditions were as follows: AmpliTaq Gold, UP Enzyme Activation 95 °C for 10 min, hold cycles, denature 95 °C for 15 s, anneal/extend 60 °C for 1 min for 40 cycles.

### 2.3. In Silico Analysis of the Four Variants of the MSH2 and PMS2 Genes

For in silico analysis, the hg38 version of the human genome and the open-access platform SpliceAI were used (available at SpliceAI Lookup https://spliceailookup.broadinstitute.org/, accessed on 20 August 2024), which allowed for the analysis of specific variants with two splicing prediction models, SpliceAI [17] and Pangolin [18]. These analyses yielded delta scores ranging from 0 to 1, and these scores can be interpreted as the probability that the variant affects splicing. The SpliceAI delta score indicates the possible gain or loss of acceptor or donor sites; additionally, the Pangolin delta score indicates possible gain or loss of splicing.

The UCSC Genome Browser (available at https://genome.ucsc.edu/index.html, accessed on 20 August 2024) was used to predict whether the variants were located in motif consensus for transcription factors (TFs), included only in the JASPAR database [19,20]. The UCSC platform utilizes a probability scale from 0 to 1, where 0 indicates low probability and 1 indicates high probability of the TFs recognizing the base at that position. In addition, TRRUST (https://www.grnpedia.org/trrust/, accessed on 20 August 2024) is a manually curated database of human transcriptional regulatory networks; it was used to explore the interactions of TFs with the genes of interest in this study [21].

### 2.4. Statistical Analysis

The distribution of quantitative data was assessed using the Kolmogorov–Smirnov test. To compare the means of variables with a normal distribution, Student’s *t*-test was utilized, whereas the Mann–Whitney U test was employed for variables that did not follow a normal distribution. The chi-square test was used to compare qualitative variables.

Genotypic and allelic frequencies were determined by direct counting. To compare genotypic and allelic distributions and the Hardy–Weinberg equilibrium, the chi-square test was used.

For the association analysis, the risk of the alleles and genotypes of *MSH2* and *PMS2* was estimated using the odds ratio (OR) with 95% confidence intervals (CI).

Haplogroups were inferred with a Bayesian algorithm. Linkage disequilibrium (LD) was measured with the Arlequin v3.5.2 software in terms of D’; r^2^ values > 0.33 were considered significant [22].

All statistical analyses were performed with SPSS v29.0.2.0. The *p*-values < 0.05 were considered significant.

## 3. Results

### 3.1. Descriptive Characteristics of the Study Groups

The general characteristics of all individuals are shown in Table 2.

### 3.2. Analysis of Genotypic, Allelic and Haplogroup Frequencies and Analysis Association

The genotypic and allelic frequencies of the four SNVs in both groups studied are shown in Table 3. The association analysis, under classical models of inheritance (dominant, recessive, codominant or additive) did not reveal genotypes or alleles of *MSH2* and *PMS2* genes associated with risk for CRC (*p* > 0.05) (Table 3).

The haplogroups were constructed in the following order: rs2303426, rs10179950, rs2286681 and rs62456178. Fifteen different haplogroup combinations were detected. The four most frequent were CCAG, GTAG, GTCA and CCCA in both groups (Table 4).

Additionally, LD was observed for loci rs2303426 and rs10179950 in *MSH2* in both CRC (r^2^ = 0.7143, D’ = 0.8571; *p* < 0.0001) and reference (r^2^ = 0.8674, D’ = 0.9442; *p* < 0.0001) groups, and also for loci rs2286681 and rs62456178 in *PMS2* in patients (r^2^ = 0.7027, D’ = 0.9323; *p* < 0.0001) and reference subjects (r^2^ = 0.6295, D’ = 0.9313; *p* < 0.0001).

### 3.3. In Silico Analysis of the Four Studied Variants

Finally, the in silico analysis revealed that none of the four examined variants impact on splicing, as the delta scores with SpliceAI were <0.13 and the delta scores in Pangolin were <0.03 for all four SNVs. The analyses of motif consensus for TFs revealed that rs2303426 is located within the recognition sequence for ZNF530 and ZFP14, which both showed a high probability for the reference C allele. For rs2286681, a total of 33 transcription factors (TFs) that recognize the antisense strand were identified, with NFE2 and FOS being particularly notable because the allele at the variant position was highly conserved within the TF consensus motif, with a value of 1.0. The positions of rs10179950 and rs62456178 are not located in recognition sites for any transcription factors. Furthermore, according to TRRUST, these transcription factors were not found in the regulatory network with the *MSH2* or *PMS2* gene.

## 4. Discussion

The mean age of 58.2 ± 14.7 years found in the group with CRC agrees with the mean age (59.06 ± 13.48 years) reported in a comparable study of 180 Mexican patients also with CRC (*p* = 0.5847) [23]. These mean ages differ from what is reported globally (65–74 years) [24] and suggest differences in the corresponding risk factors for CRC across diverse populations. The 2018–2019 National Health and Nutrition Survey in Mexico revealed a high prevalence of predisposing factors for CRC such as overweight, obesity and diabetes, which could explain the earlier diagnosis of CRC in Mexican individuals compared with other populations [25].

A slight increase in the percentage of male patients (60.8%) in the present study is consistent with the fact that CRC is more common in men than in women [26]. This gender disparity can be attributed to risk factors associated with CRC. Men tend to have less healthy diets than women, with a higher consumption of red meat and lower fiber intake. Additionally, men consume more alcohol and tobacco than women [27] while estrogen might confer a protective effect against the development of CRC in women [28].

The weight and BMI of patients with CRC were significantly lower than those of the reference group (*p* < 0.0001 for both values). These findings could reflect the weight loss due to factors such as decreased caloric intake, chronic inflammation and impaired metabolism of glucose, proteins and lipids in cancer patients [29]. Additionally, chemotherapy often causes nausea and vomiting, which worsen weight loss [30].

The lack of significant differences between the study groups regarding the variants rs2303426 C>G and rs10179950 C>T of *MSH2* concurs with previous reports that failed to link them to CRC. Moreover, no association of the rs2303426 variant with squamous cell carcinoma of the head and neck was found [31]. In contrast, other authors [12] reported a significant association of the rs2303426CC genotype with lung cancer in 500 patients who had a doubled risk of suffering late stages of the disease (OR = 1.97, CI = 0.99–3.94, *p* = 0.03). Additionally, the alternate allele conferred a 2.25-fold greater risk of lymph node invasion compared with the reference allele (OR = 2.25, CI = 1.20–4.23, *p* = 0.01).

Similarly, Srivastava et al. [13] demonstrated that homozygous genotypes of the rs2303426 variant entail a significant risk of developing gallbladder cancer (OR = 1.83, *p* = 0.052). In another report [32], the rs10179950 C>T variant of *MSH2* was associated with a protective effect against the development of breast cancer in different populations (OR = 0.89, CI = 0.81–0.97, *p* = 0.0085).

For the variants rs2286681 A>C and rs62456178 G>A in *PMS2*, no significant differences were found between the study groups. However, the rs2286681A allele was associated with an increased risk of developing lung cancer (OR = 1.16, CI = 0.70–1.91, *p* = 0.034) and head and neck cancer (OR = 1.42, CI = 0.90–2.24, *p* = 0.021) [16]. These discrepant findings may be due to differences in the specific genetic structure of each population [33]. As for the variant rs62456178, there are no reports on its potential relationships with any type of cancer.

In this study, the haplogroups constructed with the four variants (rs2303426, rs10179950, rs2286681A and rs62456178) in the *MSH2* and *PMS2* genes are reported for the first time in the literature. The distribution of haplogroup frequencies in both groups, patients with CRC and reference subjects, was similar (*p* > 0.05). The LD observed in both pairs of loci in the two study groups suggests that they segregate together rather than independently.

In the in silico analysis, the absence of repercussions on post-replicative processing may be because all four variants studied are in central areas of the introns and consequently do not interfere with the consensus splicing regions. With respect to the analysis of predicted TF binding sites for rs2303426 and rs2286681, it was found that the reference allele exhibited a higher affinity for recognition compared with the alternative allele. This suggests that if the variant was present, the transcription factor would bind less strongly or not at all, potentially resulting in a decreased transcriptional rate.

Based on the results obtained in this study, no association between the variants and CRC has been identified, suggesting that these variants do not have clinical, physiological or biological implications for the patients. However, the potential effect of these variants on protein regulation remains undefined, despite the in silico analysis results. Therefore, a functional study could be conducted subsequently to explore how changes in the DNA sequence might influence gene expression regulation or protein production.

Finally, the main limitation of our study is the scarce information regarding the four SNVs, which prevents a broader discussion of the results obtained, as well as the unreliable statistical data in previous reports featuring a CI that includes 1 and *p* > 0.05.

## 5. Conclusions

The genotypic and allelic frequencies of the rs2303426 and rs10179950 variants in the *MSH2* gene and the rs2286681A and rs62456178 variants in the *PMS2* gene are reported for the first time in a Mexican population with CRC. While no association was found between these variants and the risk for CRC, a strong LD was observed between the pair of loci in each gene. None of the variants showed a possible repercussion on splicing.

## Figures and Tables

**Table 1:** Assay ID TaqMan^®^ SNP Genotyping.

Gene	SNV	Assay ID
*MSH2*	rs2303426	C__11796409_20
	rs10179950	C__11796411_10
*PMS2*	rs2286681	C__16180310_10
	rs62456178	C__88836525_10

**Table 2:** General characteristics of the CRC patients and reference group.

Characteristic		CRC Patients	Reference Group	
		*n* = 143	*n* = 146	
		X ± SD or %	X ± SD or %	*p*-Values
Age (years)		58.2 ± 14.7	35.9 ± 11.5	*p* < 0.0001 *
Height (m)		1.65 ± 0.10	1.66 ± 0.09	*p* = 0.3721 **
Weight (kg)		67.89 ± 14.97	78.57 ± 15.67	*p* < 0.0001 *
BMI (kg/m^2^)		25.42 ± 5.25	28.24 ± 4.58	*p* < 0.0001 **
Gender	Male	60.8	59.6	*p* = 0.8281 ***
	Female	39.2	40.4	

X: average; SD: standard deviation; m: meters; kg: kilograms; BMI: bone mineral index. * Student’s *t*-test; ** Mann–Whitney U test; *** chi-square test.

**Table 3:** Genotype and allele distributions of the studied variants in the *MSH2* and *PMS2* genes in the CRC patients and reference group.

		CRC Patients		Reference Group			
	Genotype or Allele	*n* = 143		*n* = 147			
Gene SNV		*n*	%	*n*	%	*p*-Value	OR (95% CI)
*MSH2* rs2303426	CC	44	30.8	42	28.8		1.0 (Reference)
	CG	67	46.8	71	48.6	0.703	0.901 (0.526–1.544)
	GG	32	22.4	33	22.6	0.814	0.926 (0.486–1.763)
	C	155	54.2	155	53.1		1.0 (Reference)
	G	131	45.8	137	46.9	0.788	0.956 (0.690–1.326)
*MSH2* rs10179950	CC	45	31.5	41	28.1		1.0 (Reference)
	CT	66	46.1	74	50.7	0.449	0.813 (0.475–1.391)
	TT	32	22.4	31	21.2	0.853	0.941 (0.491–1.802)
	C	156	54.5	156	53.4		1.0 (Reference)
	T	130	45.5	136	46.6	0.786	0.956 (0.689–1.326)
*PMS2* rs2286681	AA	68	47.5	66	45.2		1.0 (Reference)
	AC	53	37.1	59	40.4	0.592	0.872 (0.528–1.441)
	CC	22	15.4	21	14.4	0.962	1.017 (0.511–2.022)
	A	189	66.1	191	65.4		1.0 (Reference)
	C	97	33.9	101	34.6	0.864	0.971 (0.688–1.369)
*PMS2* rs62456178	GG	56	39.2	49	33.6		1.0 (Reference)
	GA	64	44.7	71	48.6	0.362	0.789 (0.473–1.315)
	AA	23	16.0	26	17.8	0.459	0.774 (0.392–1.527)
	G	176	61.5	169	57.9		1.0 (Reference)
	A	110	38.5	123	42.1	0.369	0.859 (0.616–1.198)

SNV: single nucleotide variant; OR: odds ratio; CI: confidence interval.

**Table 4:** Haplogroup frequency by study groups.

	CRC Patients *n* = 143	Reference Group *n* = 146
Haplogroup	*n* (Frequency)	*n* (Frequency)
C C A G	42 (0.296)	45 (0.306)
G T A G	36 (0.253)	35 (0.238)
G T C A	20 (0.140)	25 (0.172)
C C C A	25 (0.176)	23 (0.158)
C C A A	5 (0.034)	6 (0.047)
G T A A	4 (0.026)	5 (0.040)
G C A G	5 (0.036)	2 (0.012)
C T A G	3 (0.016)	1 (0.008)
C C C G	0	1 (0.009)
C T C A	1 (0.009)	0
C T C G	1 (0.007)	0
G C C G	1 (0.007)	0
G T C G	0	1 (0.005)
G C A A	0	1 (0.002)
C T A A	0	1 (0.002)

## Data Availability

The original contributions presented in the study are included in the article, further inquiries can be directed to the corresponding author.
